# Bi-insular cortical involvement in anti-NMDA-receptor encephalitis – a case report

**DOI:** 10.1186/s12883-016-0653-9

**Published:** 2016-08-08

**Authors:** Harald Hegen, Christian Uprimny, Astrid Grams, Irene Virgolini, Melanie Ramberger, Ronny Beer, Raimund Helbok, Bettina Pfausler, Erich Schmutzhard

**Affiliations:** 1 Department of Neurology, Medical University of Innsbruck, Anichstrasse 35, A-6020 Innsbruck, Austria; 2 Department of Nuclear Medicine, Medical University of Innsbruck, Innsbruck, Austria; 3 Department of Neuroradiology, Medical University of Innsbruck, Innsbruck, Austria; 4 Neuroimmunology Laboratory, Department of Neurology, Medical University of Innsbruck, Innsbruck, Austria

**Keywords:** NMDA receptor, Autoimmune encephalitis, Insular cortex, Hippocampus, Medial temporal lobe, Frontal lobe, MRI, PET, Case report

## Abstract

**Background:**

Anti-NMDA-receptor encephalitis is an immune-mediated inflammatory disorder of the central nervous system. Brain MRI is unremarkable in at least 50 % of patients and highly variable in the remaining patients with signal abnormalities in different brain regions. Only scarce reports exist on other imaging modalities.

**Case presentation:**

A 31-year-old woman sub-acutely developed psychosis, behavioural changes, amnesia, alternating states of agitation and mutism, fever and epileptic seizures. Clinically suspected diagnosis of anti-NMDA-receptor encephalitis was confirmed by the detection of anti-NMDA receptor antibodies in CSF and serum. During the acute phase, brain MRI abnormalities were found in both insular cortices and hippocampi, whereas F^18^-FDG-PET showed hypermetabolism bilaterally in insular and prefrontal cortex. After resection of the underlying ovarian teratoma and with multimodal immunotherapy the patient substantially improved reaching a modified Rankin Scale score of 2 after 3 months. At follow-up, both hippocampi were still affected on MRI, whereas insular cortex appeared normal; however, both regions showed prominent glucose hypometabolism.

**Conclusions:**

Here, we report bi-insular cortical abnormalities on MRI and F^18^-FDG-PET in a patient with anti-NMDA-receptor encephalitis during the acute phase and after clinical improvement.

## Background

Anti-N-methyl-D-aspartate-receptor (NMDAR) encephalitis is an immune-mediated inflammatory disorder of the central nervous system first described in 2007 [1]. Patients usually present with a clinical syndrome including psychosis, behavioural changes, amnesia and epileptic seizures, frequently followed by dyskinesia and decreased levels of consciousness [2]. The disease has a female predominance and is associated with an underlying tumour, mostly ovarian teratoma, in approximately 50 % of cases [2]. Patients are treated with tumour resection if applicable and immunotherapy (corticosteroids, intravenous immunoglobulins and/ or plasma exchange, and in case of treatment failure cyclophosphamide and/ or rituximab) [3]. About 75 % of patients fully recover or show mild disability, the remaining patients suffer from severe disability or die. The main predictor of good outcome is the early initiation of treatment [3].

Diagnosis is obtained by the detection of anti-NMDAR antibodies in cerebrospinal fluid (CSF) and/ or serum [4]. Besides that, diagnostic work-up includes further examinations such as routine CSF analysis, tumour screening and brain imaging [2]. Brain MRI is unremarkable in 50–90 % of patients [2, 3, 5]; in the remaining patients abnormalities are highly variable evolving in the white and grey matter [6]. White matter lesions have been reported in the medial temporal, frontal, parietal, occipital lobe [1, 4, 5, 7, 8], cingulate gyrus [9, 10] and corpus callosum [4], whereas grey matter has been shown to be involved in cerebral cortex [1, 4, 9] and certain nuclei such as thalamus [11] or basal ganglia [4, 12]. In addition, affections of the cerebellum [4, 9, 13] and brainstem [4, 9] have been observed. MRI alterations are typically subtle despite the severity and duration of signs and symptoms [2].

Only scarce evidence exists on other imaging modalities. F^18^-FDG-PET can reveal pathological changes even when MRI is normal [6]. Glucose hypermetabolism has been found in frontotemporal areas [5, 8–10, 14], in the cerebellum [5, 8, 9], brainstem [9], thalamus and basal ganglia [8].

In the following, we present a case of anti-NMDAR encephalitis with bi-insular cortical abnormalities on MRI and F^18^-FDG-PET during the acute phase of the disease and after substantial clinical improvement.

## Case presentation

A 31-year-old woman presented at the hospital’s emergency department with prominent, sub-acutely evolving psychiatric signs and symptoms including bizarre behaviour, delusional thoughts and alternating states of agitation and mutism. Besides that, she showed short-term amnesia, fever and complex epileptic seizures. Her family reported that she had already complained about insomnia and general discomfort for the past 2 weeks; otherwise there was no history of any diseases and no use of concomitant medication. The neurological examination revealed no further abnormalities, especially no meningism and no focal deficits.

**Fig. 1:**
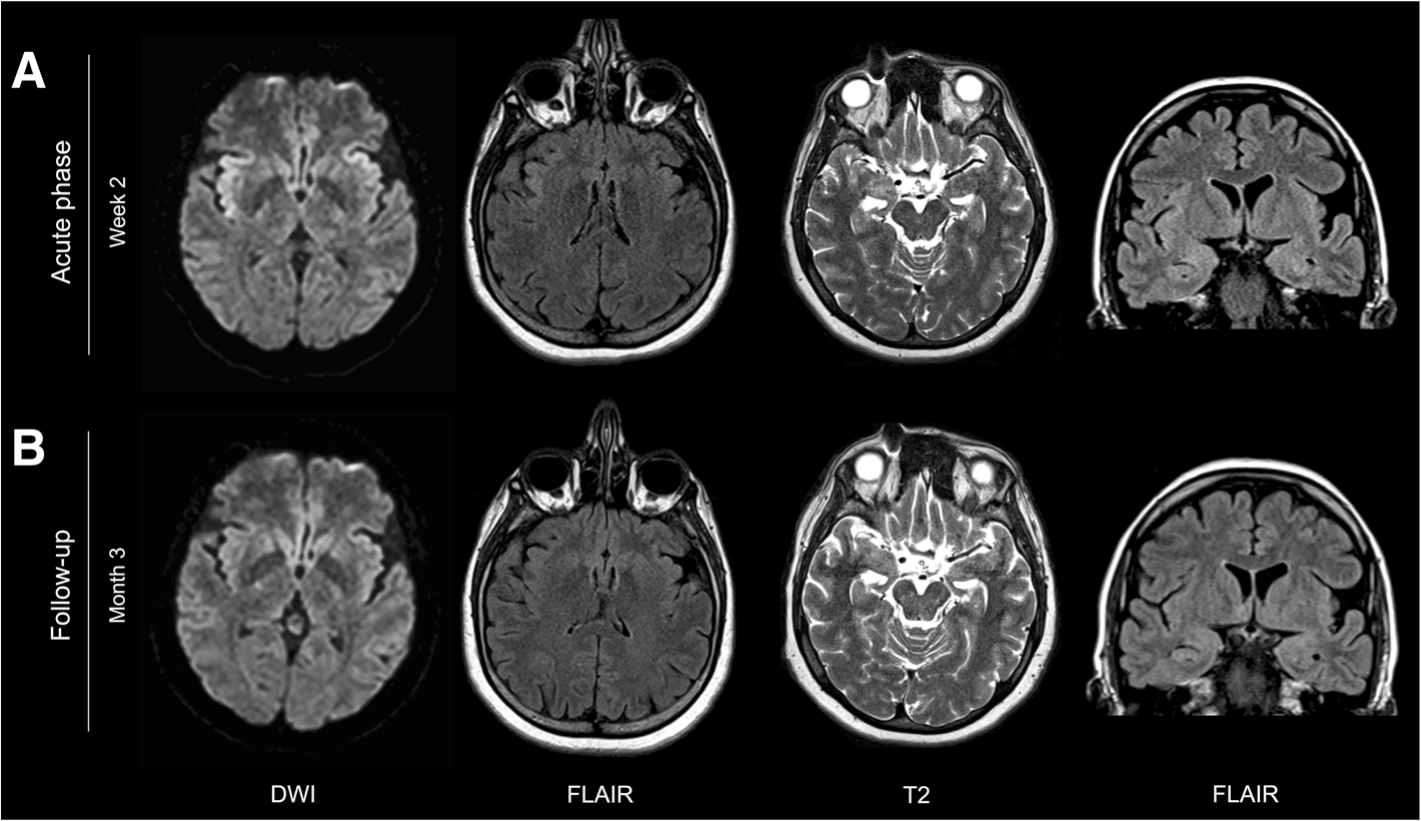
Insular and hippocampal MRI abnormalities. **a** MRI during acute phase of the disease (2 weeks after onset) shows diffusion restriction on diffusion-weighted images (DWI), hyperintensity on fluid-attenuated inversion recovery (FLAIR) images bilaterally, right-accentuated in the insular cortex as well as slight hyperintensity on T2-weighted and FLAIR images in both hippocampi. **b** After 3 months with substantial clinical improvement bi-insular diffusion restriction and FLAIR abnormalities have disappeared; hippocampal T2/ FLAIR hyperintensities are still visible

**Fig. 2:**
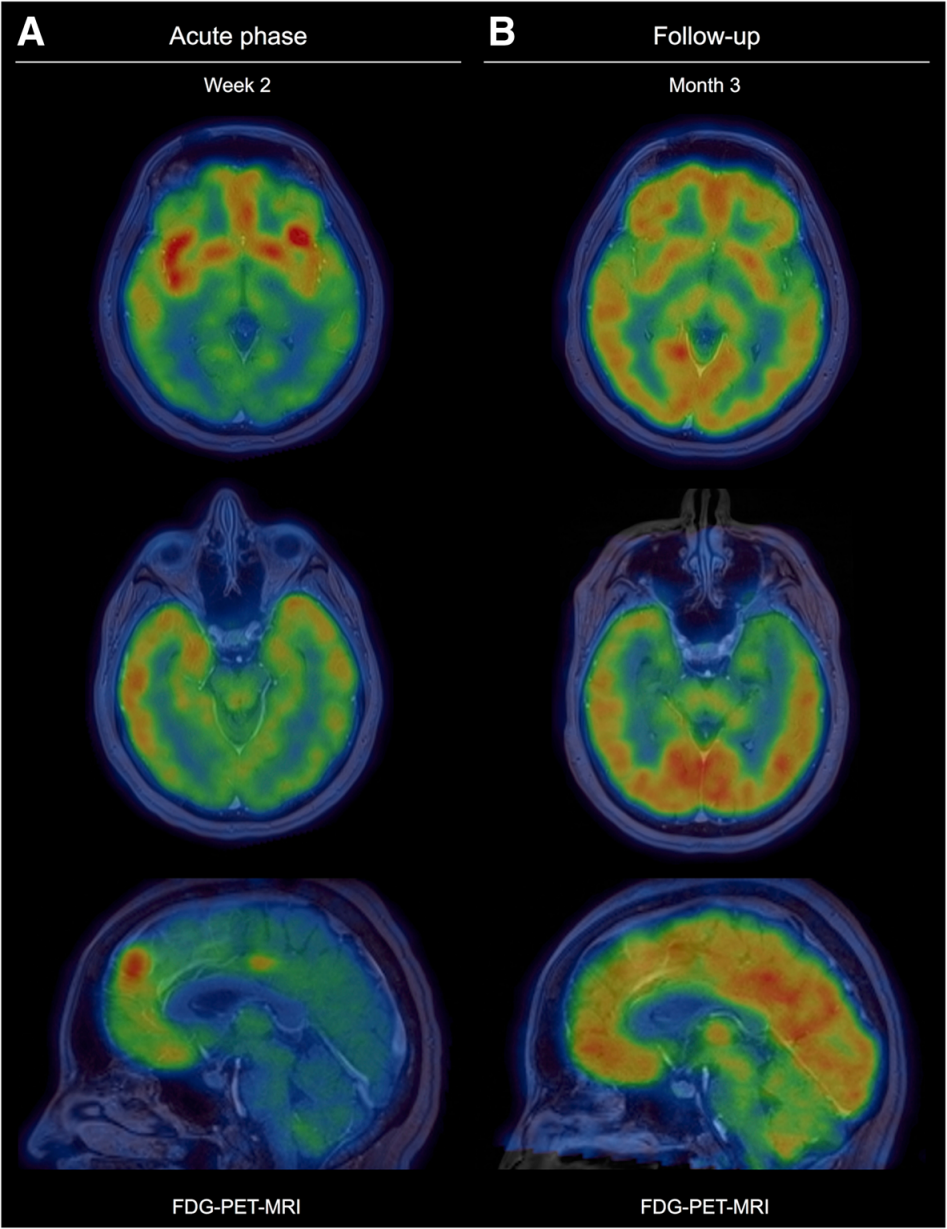
Evolution of glucose metabolism over time. **a** F^18^-FDG-PET during acute phase of disease (2 weeks after onset) shows bi-insular and bi-frontal glucose hypermetabolism as well as glucose hypometabolism occipital and partially in the parietal lobe. **b** After 3 months there is a marked bi-insular and bi-hippocampal hypometabolism, whereas prefrontal, occipital and parietal glucose-uptake is normal. F^18^-FDG-PET scans have been co-registered with MRI in order to correlate metabolic pathologies with anatomical structures

Emergency brain MRI showed diffusion-restriction on diffusion-weighted images in insular cortex, and hyperintensity on fluid-attenuated inversion recovery (FLAIR) and T2-weighted images in insular cortex and hippocampus (Fig. 1a). F^18^-FDG-PET showed hypermetabolism in insular and prefrontal cortex (Fig. 2a). CSF analysis revealed a mild pleocytosis comprising mononuclear cells. Diagnosis was made by the detection of anti-NMDAR antibodies in CSF and serum. Tumour screening by whole body imaging revealed a tumour of the right ovary that was subsequently resected and histopathologically proven to be a teratoma.

After tumour resection and multimodal immunotherapy (plasmapheresis for 4 days followed by intravenously applied corticosteroids over 5 days at a total dose of 4 g) the patient improved substantially. In serum samples, anti-NMDAR antibody titres decreased from 1:160 to 1:20 [15]. After 3 months the patient still had slight disability, was able to look after her own affairs without assistance, but was unable to carry out all previous activities, mainly due to memory deficits (corresponding to a modified Rankin Scale score of 2). At this point of time insular abnormalities on diffusion- and T2/ FLAIR-weighted MRI had disappeared, whereas hippocampal FLAIR/ T2-hyperintensity remained (Fig. 1b). In both areas prominent glucose hypometabolism was observed; glucose-metabolism in prefontal cortex was normal (Fig. 2b).

## Conclusions

Here, we report bi-insular cortical involvement in anti-NMDA-receptor encephalitis [6] as shown by structural and metabolic abnormalities. The striking correlation between initial MRI findings (T2/ FLAIR hyperintensity and diffusion-restriction, respectively) and initial F^18^-FDG-PET hypermetabolism that was followed by a prominent hypometabolism suggests that structural disruption and not only functional changes in brain metabolism results in medium-term brain dysfunction [16].

There are some considerations with regard to different, longitudinally performed imaging modalities in patients with anti-NMDAR encephalitis. In general, imaging abnormalities reflect a present disruption within brain areas, which might improve [16] or even disappear with clinical improvement, as this was the case in our patient. However, it has to be highlighted that a significant proportion of patients shows normal routine MRI during the acute phase of the disease and that structural changes might only be detected by more sophisticated MRI methods such as volumetry, analysis of microstructural integrity or resting state functional connectivity [17, 18]. Timing of imaging is another crucial issue. Whereas MRI abnormalities might resolve over time, hypermetabolic areas on F^18^-FDG-PET during the acute phase of the disease typically turn into hypometabolic state during the early recovery phase. During this time, patients have already clinically improved but are still disabled, as this was also the case in our patient. After a longer follow-up glucose hypometabolism might be alleviated and accompanied by further clinical improvement [19].

In conclusion, this case confirms that there exists a serial changing brain pattern on MRI and F^18^-FDG-PET and depicts involvement of insular cortex in patients with anti-NMDA-receptor encephalitis.

## Abbreviations

CSF, cerebrospinal fluid; F^18^-FDG-PET, 18-Fluoro-deoxyglucose positron emission tomography; MRI, magnetic resonance imaging; NMDAR, N-methyl-D-aspartate-receptor
